# Toward a stable and low-resource PLM-based medical diagnostic system via prompt tuning and MoE structure

**DOI:** 10.1038/s41598-023-39543-2

**Published:** 2023-08-03

**Authors:** Bowen Dong, Zhuo Wang, Zhenyu Li, Zhichao Duan, Jiacheng Xu, Tengyu Pan, Rui Zhang, Ning Liu, Xiuxing Li, Jie Wang, Caiyan Liu, Liling Dong, Chenhui Mao, Jing Gao, Jianyong Wang

**Affiliations:** 1 Department of Computer Science and Technology, Tsinghua University, Beijing, China; 2 Department of Neurology, State Key Laboratory of Complex Severe and Rare Diseases, Peking Union Medical College Hospital, Beijing, China; 3 School of Software, Shandong University, Jinan, China; 4 Key Laboratory of Intelligent Information Processing Institute of Computing Technology, Chinese Academy of Sciences (ICT/CAS), Beijing, China; 5 University of Chinese Academy of Sciences, Beijing, China

**Keywords:** Data mining, Machine learning, Alzheimer's disease

## Abstract

Machine learning (ML) has been extensively involved in assistant disease diagnosis and prediction systems to emancipate the serious dependence on medical resources and improve healthcare quality. Moreover, with the booming of pre-training language models (PLMs), the application prospect and promotion potential of machine learning methods in the relevant field have been further inspired. PLMs have recently achieved tremendous success in diverse text processing tasks, whereas limited by the significant semantic gap between the pre-training corpus and the structured electronic health records (EHRs), PLMs cannot converge to anticipated disease diagnosis and prediction results. Unfortunately, establishing connections between PLMs and EHRs typically requires the extraction of curated predictor variables from structured EHR resources, which is tedious and labor-intensive, and even discards vast implicit information.In this work, we propose an Input Prompting and Discriminative language model with the Mixture-of-experts framework (IPDM) by promoting the model’s capabilities to learn knowledge from heterogeneous information and facilitating the feature-aware ability of the model. Furthermore, leveraging the prompt-tuning mechanism, IPDM can inherit the impacts of the pre-training in downstream tasks exclusively through minor modifications. IPDM remarkably outperforms existing models, proved by experiments on one disease diagnosis task and two disease prediction tasks. Finally, experiments with few-feature and few-sample demonstrate that IPDM achieves significant stability and impressive performance in predicting chronic diseases with unclear early-onset characteristics or sudden diseases with insufficient data, which verifies the superiority of IPDM over existing mainstream methods, and reveals the IPDM can powerfully address the aforementioned challenges via establishing a stable and low-resource medical diagnostic system for various clinical scenarios.

## Introduction

Health is one of the major concerns of humanity all the time and a vital factor affecting human survival and development. However, diseases especially chronic diseases with inconspicuous early-rising features and sudden diseases with insufficient data, have gradually become the greatest threat to human health. With the emerging branch of the medical domain and the maturity of life science and technology, considerable effective therapeutic schedules have been proposed and time-tested. Numerous disease threats have been gradually conquered. Nevertheless, there are still some intractable diseases (such as Alzheimer’s disease) or extremely urgent situations (such as emergencies in ICU) that are challenging for medical institutions to provide timely treatment with imperceptible early symptoms and insufficient features. Even in places with sufficient medical resources, due to the exorbitant cost of testing, many intractable diseases are not always discovered until it threatens the patients’ health when the critical period for intervention and treatment has already been missed.

Moreover, doctors with relevant expertise in diagnosing complex diseases become significantly scarcer in places without sufficient medical resources. The vision of establishing a complete medical and health service system in underdeveloped regions is remarkably arduous since the extreme imbalance of medical resources between different regions further aggravates the difficulty of disease prediction and diagnosis.

The past decade has witnessed the prominent development of machine learning, and machine learning methodology has achieved tremendous success in various downstream application areas, such as assistant disease diagnosis and prediction^1–6^, autonomous driving^7,8^ and stock market prediction^9,10^. Benefiting from the rapid development of machine learning techniques, machine learning models have gradually become the prevalent approach to mitigate pressing the above problems via reducing medical costs and deeply mining implicit information^11–13^. However, these models typically require quantities of training data as the prerequisite, and the performance of existing models will suffer an unendurable decline when annotated data is insufficient, which significantly restricts the applicability of these methods^14,15^. Unfortunately, the number of annotated data remains far from adequate to facilitate the various real-world application requirements, especially in medicine and relevant fields^16–18^.

Therefore, transfer learning is proposed. Pre-trained language models (PLMs), which are currently the most popular transfer learning models, divide the training into two phases: pre-training and fine-tuning, pre-training on a large-scale open-domain corpus and fine-tuning on downstream tasks^19–23^. PLMs compensate for the negative effects of insufficient training data by transferring pre-training results to downstream tasks and have achieved impressive success in natural language processing (NLP) tasks^24–29^. However, PLMs are usually pre-trained on natural language corpus, which has a natural gap with the most commonly used structured electronic health records (EHRs) in disease diagnosis and prediction tasks^30–33^. Although there have been works like Med-BERT^34^ and BEHRT^33^ to rearrange the pre-training task for structured EHRs, the large-scale data and the expensive training cost required for pre-training make it suffer from various deficiencies. Nowadays, the parameter scale of PLMs has reached trillions, and the number of GPU hours that pre-training needs has reached millions. Reconstructive pre-training is not only a massive waste of computing resources but also an unacceptable delay in the application of advanced models in the medical field.

In order to solve the above problems, we propose IPDM (Input Prompting and Discriminative language model with the Mixture-of-experts framework), which can penetrate the pre-training knowledge of PLMs to structured EHRs with relatively minor modifications in downstream tasks. Our insight to tackle the challenges mentioned above is to design a stable and low-resource medical diagnostic infrastructure system via PLMs, which can assist medical experts in conducting auxiliary analysis. Preliminary, the input prompt constructed with meta information is used as the input of the system. Moreover, the models in the system use the prompt-tuning method based on the discriminant pre-training language models. Eventually, according to the gating network, the prediction of the multiple models with the same structure but different initialization are weighted as the output.

The effectiveness of IPDM was evaluated by fine-tuning one disease diagnosis task and two disease prediction tasks: Alzheimer’s disease diagnosis task, Alzheimer’s disease progression prediction task, and ICU death prediction task. On these three tasks, comprehensive experiments were carried out with Logistic Regression (LR)^35^, Support Vector Machine (SVM)^36^, Decision Tree (DT)^37^, Random Forest (RF)^38,39^, Multi-layer Perceptron (MLP)^40^, Convolutional Neural Network (CNN)^41–43^ and Long Short Term Memory (LSTM)^44^ to prove the effectiveness of IPDM. In order to verify the effectiveness of IPDM in the case of imperceptible early symptoms and insufficient features, we designed the few-feature setting, and we designed the few-sample setting to verify whether the performance of IPDM will be significantly affected under low-resource scenarios. Moreover, we also verify the effectiveness of different improvements in IPDM through ablation experiments.

Our main contributions are summarized as follows:

1. Instead of reconstructive pre-training, IPDM transforms the pre-training results from natural language pre-training corpus to structured EHRs with minor changes on fine-tuning and outperforms other representative machine learning methods on one disease diagnosis task and two disease prediction tasks.
2. Experimental results with the few-feature setting show that IPDM is more competent than other representative machine learning methods in the diagnosis and prediction of chronic diseases with imperceptible early symptoms and sudden diseases with insufficient features.
3. Under low-resource scenarios, experimental results show that IPDM has better stability compared with other representative machine learning methods.
4. Ablation experiments demonstrate the effectiveness of different improvements in IPDM.

## Results

### Data source

Our data consist of two databases, ADNI Database and MIMIC-III Database^45,46^.

#### ADNI database

**Figure 1:**
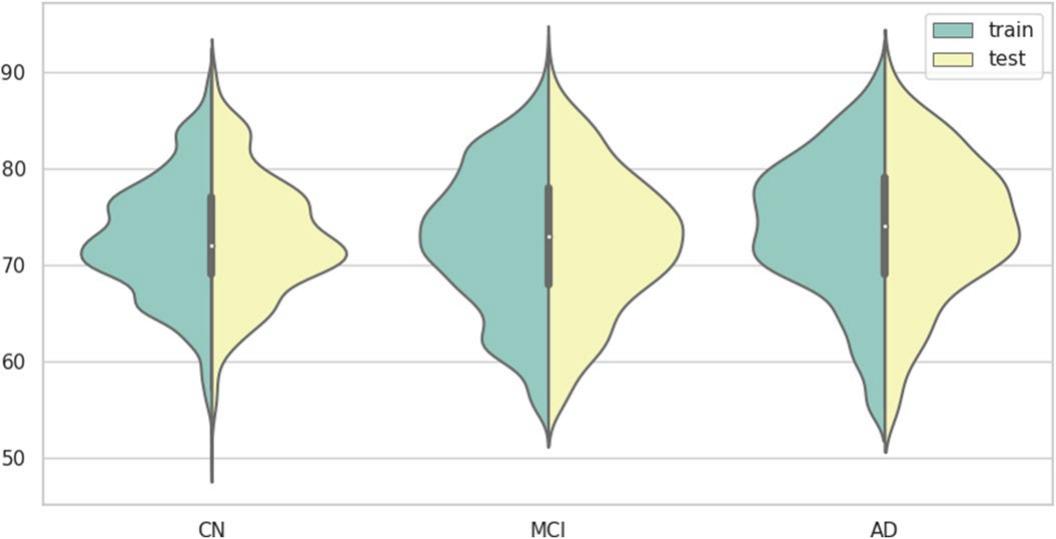
Violin distribution of subjects at different ages and different stages from ADNI database. The vertical axis is the age of the subjects, mainly distributed between 45 and 100 years old. The horizontal axis is based on whether the subjects are CN, MCI or AD. The left and right sides of each violin chart are the distribution of the training data and the test data, and the two distributions are similar.

The development of Alzheimer’s disease (AD) usually starts from the cognitive normal (CN) stage of the patient, and neurodegeneration leads to brain damage, which accumulates to a certain level and causes mild cognitive impairment (MCI)^47,48^. This stage is often accompanied by partial cognitive impairment and memory loss. After the disease continues to deteriorate and develops into AD, there will be comprehensive cognitive impairment and severe memory loss, which will eventually lead to death. Alzheimer’s Disease Neuroimaging Initiative (ADNI) is a research program for AD pathology initiated in the United States. The program has recruited more than 1500 subjects over three phases (ADNI I, ADNI II, and ADNI GO), mainly from the United States and Canada, between the ages of 55 to 100, including people with CN, MCI and AD. Figure 1 shows the distribution of subjects at different ages and different stages. During the program, the subjects’ Alzheimer’s disease-related features were recorded every 6 months, and finally, all subjects’ features were collected in the ADNI database in the form of high-dimensional data points.

#### MIMIC-III database

The Intensive Care Unit (ICU) is the front line of life-saving in hospitals, where there is only a line between life and death. It is the function of the ICU to detect the abnormal condition of the patient in time and dispatch medical resources for rescue to the greatest extent by monitoring the patient’s physical data. Recording and collecting the monitoring data is of great significance to critical care research and live saving, thus the MIMIC Database came into being. After two generations of development, the MIMIC-III Database was released in 2015, which includes admission records, disease information and health monitoring data of nearly 50,000 patients. Due to its free and open access, the MIMIC-III Database is sought after by researchers.

### Data modality

**Figure 2:**
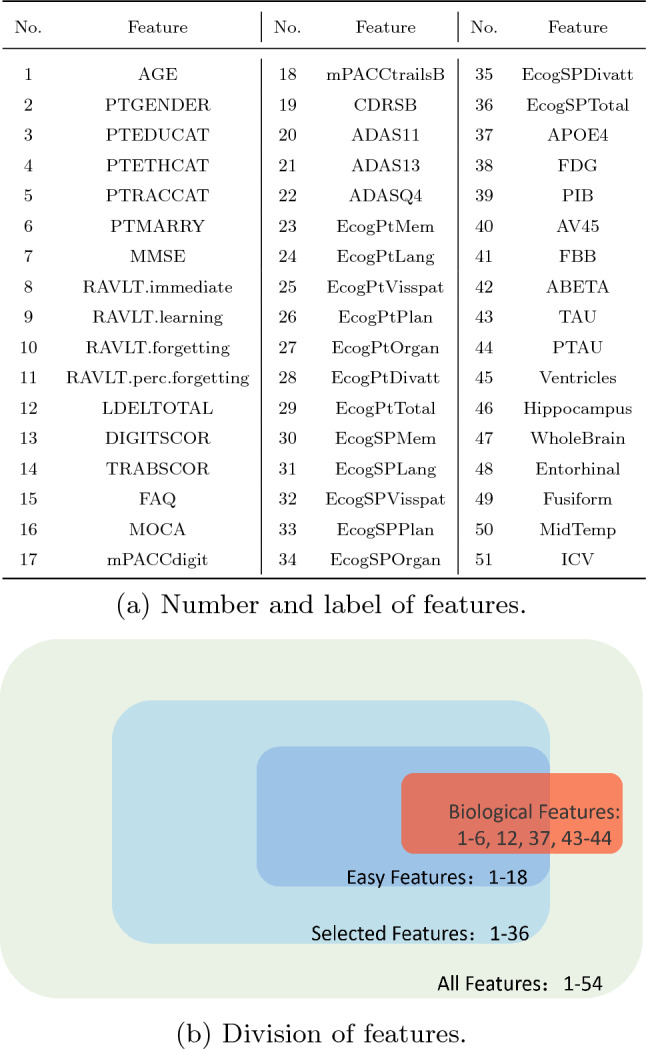
Features selected from the ADNI database. The table shows the number and label of the features, and the picture shows the division of the features, where the selected features is the proper subset of all features, and the easy feature is the proper subset of selected features.

**Figure 3:**
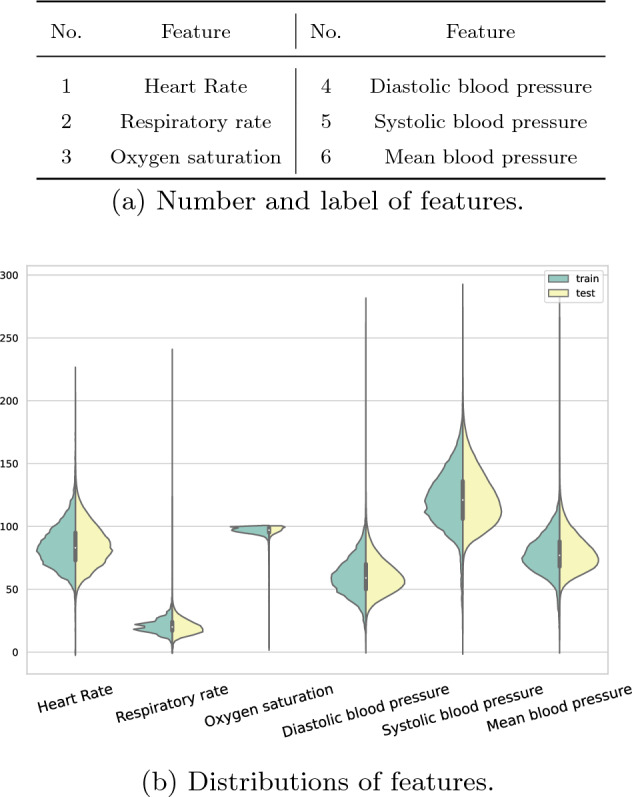
Features selected from the MIMIC-III database. The table shows the number and label of the features, and the picture shows the distribution of the features.

As shown in Figs. 2 and  3, a total of 51 features selected from ADNI database and six features selected from MIMIC-III database were used. We annotated the features from the ADNI Database as selected features, easy features and biological features. According to the suggestion from doctors, features that are not hard to collect are annotated as selected features, and the easiest to collect are annotated as easy features. TAU, P-TAU, APOE4 and demographic information are annotated as biological features.

### Experiment setting

#### Alzheimer’s disease diagnosis task(AD-D)

We collected test records and status of subjects at different times, and modeled the diagnosis of Alzheimer’s disease as a multi-classification task: according to a subject’s record, diagnose whether the subject was CN, MCI or AD?

#### Alzheimer’s disease progression prediction task(AD-P)

Records were collected from subjects who were in the MCI stage at the time of the initial test, and for the accuracy of the study, only records that continued to be tested for more than 48 months and ended up with MCI or AD were used to model Alzheimer’s disease progression prediction as a binary classification task: predict whether an MCI patient will progress to AD based on the initial test record.

#### ICU death prediction task(ICU)

The condition of patients in the ICU is extremely unstable and may deteriorate sharply at any time. Therefore, the task of predicting death in the ICU is modeled as a binary task: according to the current monitoring data (within 4 h) of a patient in ICU, predict whether the patient will die within the next 24 h.

#### Few-feature setting

ADNI database records a variety of features related to Alzheimer’s disease, but in clinical practice, it is often difficult to collect such comprehensive features due to cost and technical difficulty. To simulate this situation, few-feature setting is designed in the Alzheimer’s disease diagnosis task and progression prediction task, and models will be challenged to fine-tune only with features that are easy to acquire for diagnosis and prediction. Specifically, four different settings are used, namely All Features (All), Selected Features (Sel), Easy Features (Easy) and Biological Features (Bio), and their inclusion relationship is shown in the Fig.  2.

#### Few-sample setting

The Alzheimer’s disease diagnosis task and the ICU death prediction task have enough samples for training (as shown in Table 1), however, we can not collect so much data for some rare diseases, thus few-sample setting is designed. Models will be challenged to fine-tune with only 10% or even 1% of the training data while evaluating on the original test data.

**Table 1:** Size distribution of different task datasets Alzheimer’s disease diagnosis task (AD-D) and ICU death prediction task (ICU) have sufficient samples, but the dataset of Alzheimer’s disease progression prediction task (AD-P) is small.

Task	Training data	Test data
AD-D	8993	2000
AD-P	539	80
ICU	118,922	24,261

### Baseline

This work uses Logistic Regression (LR)^35^, Support Vector Machine (SVM))^36^, Decision Tree (DT))^37^, Random Forest (RF))^38,39^, Multi-layer Perceptron (MLP))^40^, Convolutional Neural Network (CNN))^41–43^, and Long Short Term Memory (LSTM))^44^ as the baselines. Among them, the input of LR and SVM is normalized. CNN and LSTM use the mapping of word vectors as input.

### IPDM architecture

**Figure 4:**
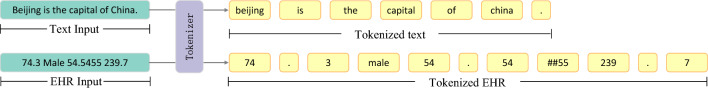
Text input and structured EHR input segmented by PLM tokenizer. In the processing of structured EHR input, “74.3” is split into “74”, decimal point and “3”, “54.5455” is split into “54”, decimal point, “54” and “## 55”, “239.7” is split into “239”, decimal point and “7”. The mapping between the natural language texts and the structured EHRs is difficult to understand.

**Figure 5:**
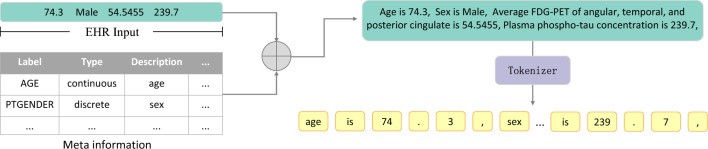
Input prompts built by meta-information. IPDM uses the descriptions of features to build input prompts.

When PLMs process natural language texts, the input is first segmented and tokenized, then the tokens are converted into embeddings according to the pre-trained vocabulary, and finally the network calculates the embeddings into probabilities^24–29^. However, since structured EHRs are heavily used in disease prediction tasks (such as the three datasets used in this work)^30,31^, the tokenizer of PLMs cannot handle it well. As shown in Fig. 4, input from the Alzheimer’s diagnosis task is segmented by the PLMs’ tokenizer, the decimal is split into at least three parts, and the long continuous number is split into multiple segments (e.g., “54.5455” is split into “54”, decimal point, “54” and “## 55”). The mapping between the natural language texts and the structured EHRs of the tokenizer of PLMs is difficult to understand^34^. And it is difficult for PLMs to distinguish table items corresponding to different values, and it is easy to produce ambiguity. IPDM introduces meta-information to build input prompts to solve this problem, as shown in Fig. 5. Meta-information refers to easily obtainable external knowledge such as labels and descriptions. Meta-information is used to convert structured EHRs into expressions closer to natural language. Input prompts can help PLMs distinguish different features and acquire some prior knowledge.

**Figure 6:**
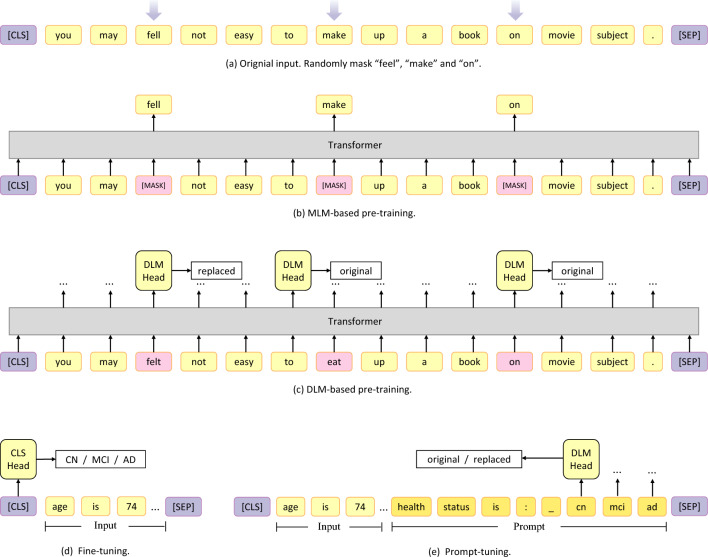
Frameworks of MLM-based pre-training, DLM-based pre-training, fine-tuning and prompt-tuning. (**a**) The original input and the word “feel”, “make” and “on” are masked randomly. (**b**) The process of MLM-based pre-training, the masked words are replaced with “[MASK]”, and the transformer regenerate them. (**c**) The process of DLM-based pre-training, the masked words are replaced by other words created by a generator. After the transformer, all words are judged by a discrimination header, named DLM-Head. In this example, the word “felt” and “eat” is judged to have be replaced and the other words are not replaced (original). (**d**) An example of fine-tuning, where the word embedding corresponding to the token “[CLS]” is used to discriminate which category this sentence belongs to. In the example of prompt tuning in (**e**), the prompt designed according to the categories is spliced after the original input, and then the discriminator is used to discriminate the tokens corresponding to the categories are replaced or not. Morever, the processes in (**c,e**) share the discrimination header (DLM Head).

The pre-training and fine-tuning paradigm of PLMs have achieved great success in NLP tasks, the most representative one is the Masked Language Models (MLMs, such as BERT, RoBERTa)^24–26^. MLMs use “[MASK]” to replace part of the words during pre-training to corrupt the input (as shown in Fig. 6a), and then train the network to regenerate the original words, which is simple and efficient, as shown in Fig. 6b. However, the disadvantage of MLMs is that the pre-training task is defined on the replaced words, which is a small subset of vocabulary, and at the same time, the absence of “[MASK]” in the downstream task leads to a natural gap between the pre-training and the fine-tuning. Discriminative Language Models (DLMs, such as ELECTRA)^27^ propose a different pre-training method: a generative network is used to generate words to replace part of the input, and the network is trained to discriminate whether the word is replaced or not. As shown in Fig. 6c, each word should be discriminated by a discrimination header, named “DLM Head”. In the example, the word “felt” is discriminated to have be replaced and the other words are not replaced (original). Compared with MLMs, DLMs define tasks on the whole vocabulary and eliminate the natural gap with downstream tasks caused by “[MASK]”, thus showing excellent results in NLP tasks.

**Table 2:** Prompts designed for different tasks. .

(a) Alzheimer’s disease diagnosis task :	Health status is : _ **cn** **mci** **ad**	
(b) Alzheimer’s disease progression prediction task :	Condition will : _ **keep** **worse**	
(c) ICU death prediction task :	Patient will : _ **live** **die**	

The categories of each task are included in the prompts. The pre-experiment results show that using task related prompts and appropriate punctuations can improve the performance.

Although DLMs have greatly narrowed the gap between pre-training and fine-tuning compared to MLMs, the gap still exist because of traditional fine-tuning strategy. Taking the classification task as an example, the traditional fine-tuning method is shown in Fig. 6d, which directly uses the embedding corresponding to the “[CLS]” at the beginning of the input to calculate the classification probability, and the discrimination header used in the pre-training process is dropped. IPDM reuses the discrimination header (DLM Head), and fine-tunes by adding prompts to the input and discriminating the prompts for classification, which we call prompt-tuning as shown in Fig. 6e. Prompt-tuning further narrows the gap and further taps the deep potential connections between pre-training corpora and downstream tasks. We first designed several prompts related to the task content for each task, and then based on our experience in NLP tasks, we added a combination of special symbols such as “,” “:”, “_”, and “#” to increase the number of templates by 18 times. Finally, we used BERT to conduct pre-experiments on all features to select stable, convergent, and highly accurate prompts. The prompts used in main experiments are shown in Table 2.

**Figure 7:**
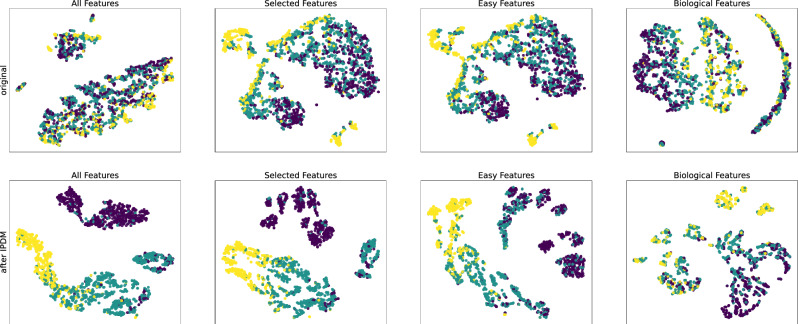
Visualize experimental results. The original data is from the test data of Alzheimer’s disease diagnosis task(AD-D) with few-feature settings. CN, MCI, and AD use purple, green, and yellow respectively. The first row is the result of using t-SNE to visualize the original data, and the second row is the result of using t-SNE to visualize the embeddings encoded by our model. Compared with the first row, the second row has obvious clustering effect. The columns from left to right are the visualize experimental results with a decreasing number of features, and the clustering effect becomes worse.

With the help of input prompts and DLM-based prompt-tuning, PLMs have been able to extract the features of structured EHR well. As shown in Fig. 7, we use t-SNE to visualize the original data of the test data of Alzheimer’s disease progression prediction task with few-feature settings, and the data points from different clusters mixed together. However, when we use t-SNE to visualize the embeddings encodered by our model, the data points of different categories have a good clustering effect. Although as the number of features decreases, the extractable information decreases, and the clustering effect of embeddings becomes worse, but it is still better than the original data.

**Figure 8:**
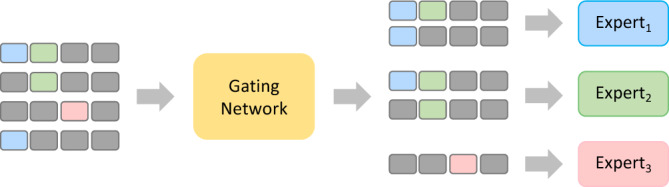
The gating network assigns samples to experts who are more sensitive to their salient features.

Structured EHRs have multiple features, and networks with different random initialization usually have different sensitivities to different features after training. Moreover, in classification tasks, different samples have different salient features. In Alzheimer’s disease progression prediction task, for example, the original data points naturally cluster into more than two categories, as shown in Fig. 7. To take better advantage of this, we adopt Mixture-of-Experts (MoE): a model is considered as an Expert, multiple experts and a trainable gating network for task assignment make up MoE^49,50^. The gating network is responsible for assigning samples to experts who are more sensitive to their salient features, as shown in Fig. 8. The original input is used by the gating network to score each expert, and then it will be constructed into input prompts with meta-information. Experts use input prompts to calculate probabilities, which are finally weighted with scores. IPDM uses a sparse gate network:

$$\begin{aligned} \begin{aligned} G(x)&=SoftMax(KeepTopK(O_i(x),K)), \\ O_i(x)&=(x\cdot W_{gate})_i+Norm((x\cdot W_{noise})_i), \end{aligned} \end{aligned}$$

where $W_{gate}$ and $W_{noise}$ are parameter-learnable matrices, $Norm(\cdot )$ is a standard normalization function, $KeepTopK(\cdot )$ keeps only the largest *K* scores, and according to the subsequent $SoftMax(\cdot )$ layer, other values are set to negative infinity here. In this way, only the derivation update of *K* experts needs to be performed, which greatly saves the calculation cost. In specific practice, limited by GPU memory, only two experts are used and set $K=1$.

For MoE, it is easy to happen that the majority of examples are assigned to a very small number of experts. If an expert is assigned a sample, the expert is said to be activated. In the most extreme case, only one expert is activated, which is no different from training only one expert, and wastes more computing resources. To avoid this from happening, the following expert-activation-balance method is used:

1. Hard constraints. Set a threshold, when an expert is activated more than this threshold, stop assigning samples to it. We set 80% of the total number of current samples as the threshold.

2. Soft constraints. Add a loss function $Loss_{Act}$ about the number of expert activations,

$$\begin{aligned} Loss_{Act} = w_{ACT} \cdot D_{KL}(Act_i\Vert Uniform), \end{aligned}$$

where $w_{ACT}$ is a hyperparameter, $Act_i$ represents the relative entropy to the uniform distribution, and $D_{KL}(\cdot \Vert Uniform)$ represents the number of activations of the i-th expert.

The more uniform the distribution of expert activations is, the smaller the loss value will be. Assuming there are *S* examples and *N* experts, then it can be approximated as:

$$\begin{aligned} Loss_{Act} = w_{ACT} \cdot \sum _i^N{ACT_i\cdot log\frac{Act_i \cdot N}{S}}_. \end{aligned}$$

### Performance boost of IPDM

**Table 3:** Experimental results compared with baselines.

Model	All Features		Selected Features		Easy Features		Biological Features		Features				100%	10%	1%
	$100\%$	$10\%$	$100\%$	$10\%$	$100\%$	$10\%$	$100\%$	$10\%$	All	Selected	Easy	Biological			
LR	76.55	75.95	78.25	76.35	75.35	73.60	55.10	52.45	80.00	81.25	78.75	76.25	78.91	78.39	77.43
SVM	75.65	62.30	80.05	75.40	77.70	73.70	52.65	44.75	82.50	${82.50}$	77.50	75.00	79.16	78.67	${78.22}$
DT	80.95	79.95	82.15	80.45	72.80	70.00	65.55	57.15	78.75	71.50	75.00	65.00	73.08	71.02	70.43
RF	${87.10}$	${85.40}$	${86.50}$	${85.10}$	77.25	${76.05}$	${67.55}$	${62.45}$	82.50	${82.50}$	${82.50}$	72.50	78.62	78.03	77.55
MLP	79.05	71.95	80.25	73.00	76.75	68.10	65.75	59.60	${83.75}$	${ {82.50}}$	${82.50}$	76.25	79.39	75.51	75.50
CNN	87.05	75.70	86.40	80.35	${ {77.80}}$	71.45	66.15	57.75	81.25	81.25	80.00	${ {77.50}}$	${ {79.91}}$	${ {79.46}}$	75.98
LSTM	84.95	62.50	85.35	62.30	77.55	60.90	67.05	55.25	75.00	72.50	70.00	67.50	79.70	78.96	76.50
IPDM	${{\textbf {87.80}}}$	${{\textbf {87.30}}}$	${{\textbf {87.55}}}$	${{\textbf {87.20}}}$	${{\textbf {80.05}}}$	${{\textbf {77.45}}}$	${{\textbf {68.60}}}$	${{\textbf {65.35}}}$	${{\textbf {88.75}}}$	${\textbf {85.00}}$	${\textbf {85.00}}$	${\textbf {80.00}}$	${{\textbf {80.70}}}$	${{\textbf {80.61}}}$	${{\textbf {79.48}}}$
$\Delta$	${+0.70}$	${+1.90}$	$+1.05$	${+2.10}$	${+2.25}$	${+1.40}$	${+1.05}$	$+2.90$	${+5.00}$	${+2.50}$	$+2.50$	$+2.50$	${+0.79}$	${+1.15}$	${+1.26}$

The three tables use the same column names. Table (a) represents the results on the Alzheimer’s disease diagnosis task, table (b) represents the results on the Alzheimer’s disease progression prediction task, and table (c) represents the results on the ICU death prediction task. The values in the table represent the accuracy. The global optimum is marked with bold, the baseline optimum is marked with an italics, and $\Delta$ represents the accuracy difference between IPDM and the baseline optimum. IPDM performs the best in all settings of all tasks and the improvements compared with the baselines are obvious.

**Table 4:** Ablation experimental results.

Model	All F.		Selected F.		Easy F.		Biological F.		Features				100%	10%	1%
	$100\%$	$10\%$	$100\%$	$10\%$	$100\%$	$10\%$	$100\%$	$10\%$	All	Selected	Easy	Biological			
IPDM	87.80	87.30	87.55	87.20	80.05	77.45	68.60	65.35	88.75	85.00	85.00	80.00	80.70	80.61	79.48
w/o Input prompt	44.75	44.75	44.75	44.75	44.75	44.75	44.75	44.75	65.00	65.00	65.00	65.00	75.43	75.43	75.43
w/o Prompt-tuning	87.65	86.35	87.50	87.15	79.15	74.30	67.60	64.55	78.75	82.50	82.50	80.00	80.47	80.66	78.40
w/o MoE	87.75	87.10	87.50	86.60	80.00	76.90	67.60	65.15	85.00	85.00	85.00	78.75	80.73	80.66	79.06
w/o Prompt-tuning &MoE	87.45	76.05	87.50	86.40	78.45	73.40	66.20	63.75	77.50	77.50	82.50	78.75	80.22	80.39	78.39

The structure of table is consistent with Table 3, “F.” is the abbreviation of “Features”. “w/o Input Prompt” means no meta-information is used to construct input prompts, “w/o Prompt-tuning” means traditional fine-tuning, “w/o MoE” means only one expert, and “w/o Promt-tuning & MoE” means traditional fine-tuning and one expert both. The results of “w/o Input Prompt” overfits the class of the highest proportion. Either “w/o Prompt-tuning” or “w/o MoE” only has a small drop in accuracy for most settings, but it becomes obvious when “w/o Promt-tuning & MoE”.

**Table 5:** Experimental results of different PLMs.

Model (w/o MoE)	All F.		Selected F.		Easy F.		Biological F.		Features				100%	10%	1%
	$100\%$	$10\%$	$100\%$	$10\%$	$100\%$	$10\%$	$100\%$	$10\%$	All	Selected	Easy	Biological			
IPDM	${\textbf {87.75}}$	${\textbf {87.10}}$	${\textbf {87.50}}$	86.60	${\textbf {80.00}}$	${\textbf {76.90}}$	${\textbf {67.60}}$	${\textbf {65.15}}$	${\textbf {85.00}}$	${\textbf {85.00}}$	${\textbf {85.00}}$	${\textbf {78.75}}$	${\textbf {80.73}}$	${\textbf {80.66}}$	${\textbf {79.06}}$
IPDM-[BERT]	87.50	86.40	87.40	86.20	${\textbf {80.00}}$	74.75	66.95	64.60	${\textbf {85.00}}$	83.75	83.75	78.25	80.50	80.51	78.50
IPDM-[RoBERTa]	87.60	86.70	${\textbf {87.50}}$	86.60	79.95	75.05	67.05	${\textbf {65.15}}$	83.75	83.25	82.50	78.25	80.66	80.22	78.40
IPDM-[ELECTRA]	87.45	76.05	${\textbf {87.50}}$	86.40	78.45	73.40	66.20	63.75	77.50	77.50	82.50	${\textbf {78.75}}$	80.22	80.39	78.39
IPDM-[BioBERT]	87.65	86.15	87.40	85.90	79.40	75.10	66.55	64.95	78.75	${\textbf {85.00}}$	82.50	${\textbf {78.75}}$	80.50	80.45	78.41
IPDM-[SciBERT]	87.30	86.60	87.15	${\textbf {86.80}}$	78.10	75.85	66.50	64.30	83.75	83.75	81.25	77.50	80.64	80.59	78.40

The structure of table is consistent with Table 4. All PlMs use only one expert. IPDM achieves the best on every setting, and it has obvious advantages in low resource scenarios.

The optim is marked with bold.

#### Comparison with baselines

**Figure 9:**
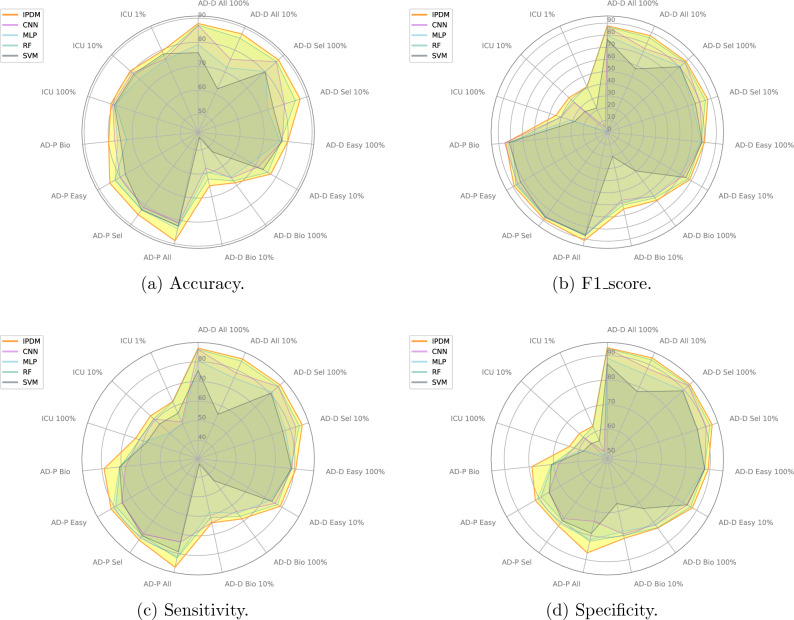
Accuracy, f1_score, sensitivity and specificity for IPDM and baselines. For the sake of clarity, we only draw SVM, RF, MLP and CNN in the figure, and the optimum (underlined) baselines under all settings come from these four baselines. Different subgraphs represent different metrics, the axis of the radar graph represents different task settings, and the length on the axis represents the value under the metric of the graph. It can be seen that IPDM almost all achieves the best under different metrics and different task settings.

The experimental results on the three tasks are shown in Table 3 and Fig. 9, where the global optimum is marked with bold, the baseline optimum is marked with an italics, and $\Delta$ represents the accuracy difference between IPDM and the baseline optimum. It can be seen that IPDM achieves the optimum under all settings, and the improvements are obvious compared with the baselines. This proves that PLMs have great potential in dealing with structured EHRs, and IPDM makes it. IPDM transforms pre-training results from natural language texts to structured EHRs with only minor changes on downstream tasks, achieving excellent results in disease diagnosis and prediction tasks.

#### Ablation experiment

In order to explore the respective contributions to the improvements in this work, ablation experiments were performed. The results are shown in Table 4, where “w/o Input Prompt” means no meta-information is used to construct input prompts, “w/o Prompt-tuning” means traditional fine-tuning, and “w/o MoE” means only one expert. Without input prompts constructed with meta-information, the model overfits the class of the highest proportion. This indicates that the input prompts play a key role when PLMs process structured EHRs, it constructs a bridge connecting natural language texts to structured EHRs. There is only a small drop in accuracy for most settings when not using prompt-tuning or using only one expert. However, when doing both, the accuracy rate drops significantly, which means that both prompt-tuning and MoE have a strong ability to stabilize the model and reduce overfitting.

#### Different PLMs

The PLM used by IPDM is the discriminative language model ELECTRA with prompt-tuning. To explore the impact of different pre-trained models, we select different PLMs from two dimensions, pre-training setting and pre-training corpus. To make the results more intuitive, we use only one expert here. From the dimension of pre-training settings, we select the most popular BERT and RoBERTa as the representatives of MLMs and select ELECTRA as the representative of DLMs. From the dimension of pre-training corpus, considering that this work is mainly aimed at the medical field, BioBERT (pre-trained on biomedical texts) and SciBERT (pre-trained on scientific texts) are selected. Unfortunately, because Med-BERT does not share its pre-trained model, and the dataset Cerner Health Facts used for pre-training has stopped permitting new users, we are not able to reproduce the work of Med-BERT and compare IPDM with it. The results of different PLMs are shown in Table 5. It can be seen that IPDM achieves the best on every setting, and it has obvious advantages in low resource scenarios. The experimental results prove that prompt-tuning improves performance by eliminating the gap between pre-training and fine-tuning.

Recently, ChatGPT (GPT-3.5) and GPT-4^51,52^ have become very popular, their powerful dialogue ability has left a deep impression on people. We attempted to directly ask the GPT models to answer diagnostic and prediction tasks without training, which we called zero-shot, the input prompts are used to chat with GPT-3.5 and GPT-4, the results are shown in the Table 6. It can be seen that the GPT models, especially GPT-4, can assist in diagnosis and prediction to a certain extent even in zero-shot situations.

**Table 6:** Experimental results of GPT-3.5 and GPT-4 with input prompt.

Task	Features	GPT-3.5	GPT-4
AD-D	All F.	60.85	71.55
	Selected F.	64.75	${\textbf {76.75}}$
	Easy F.	60.05	68.10
	Biological F.	37.80	48.85
AD-P	All F.	62.50	66.25
	Selected F.	63.75	65.00
	Easy F.	65.00	${\textbf {72.50}}$
	Biological F.	65.00	67.50
ICU		58.39	58.39

As reference, the category with the highest number in Alzheimer’s disease diagnosis task (AD-D) accounts for 44.75%, the category with the highest number in Alzheimer’s disease progression prediction task (AD-P) accounts for 65.00%, and the category with the highest number in ICU death prediction task (ICU) accounts for 58.39%.

The optim is marked with bold.

#### Low-resource scenarios

**Figure 10:**
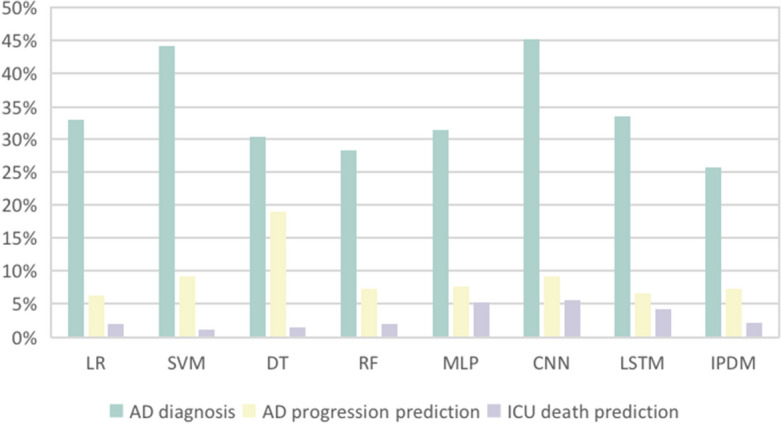
Experimental results of low-resource scenarios. The horizontal axis represents the model name, and the vertical axis represents the percentage of decrease in the worst case of the model compared with the best case under the same task. IPDM decreases less than baselines.

In this work, few-feature setting and few-sample setting are designed to simulate the real low-resource scenarios, where many models overfit and suffer an unendurable decline. Fig. 10 shows the percentage of accuracy reduction of baselines and IPDM on each task affected by low resources. It can be found that compared with the baselines, IPDM has high stability with high accuracy and is more capable of performing tasks in low-resource scenarios. This also means that IPDM can play a better role in assisting in the diagnosis and prediction of chronic diseases with imperceptible early symptoms or sudden diseases with insufficient data.

#### Implementation details

For different pre-trained language models (PLMs), we use AdamW as the optimizer. The learning rates are searched in $a \times 10^{-b}$, where $a=1$ or 5 and *b* is an integer from 1 to 7, to find the optimal for each model. We use parameters shared on huggingface.io for fine-tuning: BERT of bert-base-uncased, RoBERTa of roberta-base, ELECTRA of google/electra-base-discriminator, BioBERT of dmis-lab/biobert-base-cased-v1.2, SciBERT of allenai/scibert_scivocab_uncased. IPDM used the same pre-trained parameters with ELECTRA.

## Discussion

We have designed IPDM (Input Prompting and Discriminative language model with the Mixture-of-experts framework) to assist disease diagnosis and prediction, which achieves better results than non-pre-trained language models on one disease diagnosis task and two disease prediction tasks. IPDM is able to transform the pre-training results on natural language texts into structured EHRs by just adding input prompts to downstream tasks. We have verified the effect and necessity of input prompts through experiments on structured EHR data sets from two different databases. IPDM also uses prompt-tuning based on discriminant pre-training language models and mixture-of-experts. Ablation experiments show that these two improvements play an indispensable role in model stability and overfitting reducing. The results of few-sample setting and few-feature setting prove that IPDM has better stability and application potential in scenes lacking features and data.

**Figure 11:**
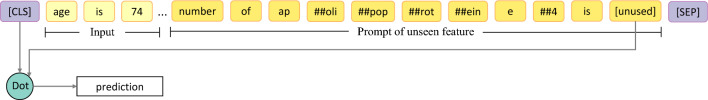
Prompts designed for unknown feature prediction experiment. The prompt information of seen features, the values of seen features, the prompt information of unseen features, and the marker words of unseen features are concatenated as the input. The dot product of the embeddings of the “[CLS]” and the marker word are used as the prediction.

In order to further explore the underlying reason why the pre-training language model has better performance, we designed an unknown feature prediction experiment. We leverage the IPDM trained on the training dataset of Alzheimer’s disease diagnosis task with a subset of features (seen features) to predict the value of features that were not used (unseen features) on the test dataset. Specifically, we only utilize one expert and freeze other experts. The prompt information of seen features, the values of seen features, the prompt information of unseen features, and the marker words of unseen features are concatenated as the input. Thereafter, the dot product of the embeddings of the “[CLS]” and the marker word are calculated as the predicted value, as shown in Fig. 11.

**Figure 12:**
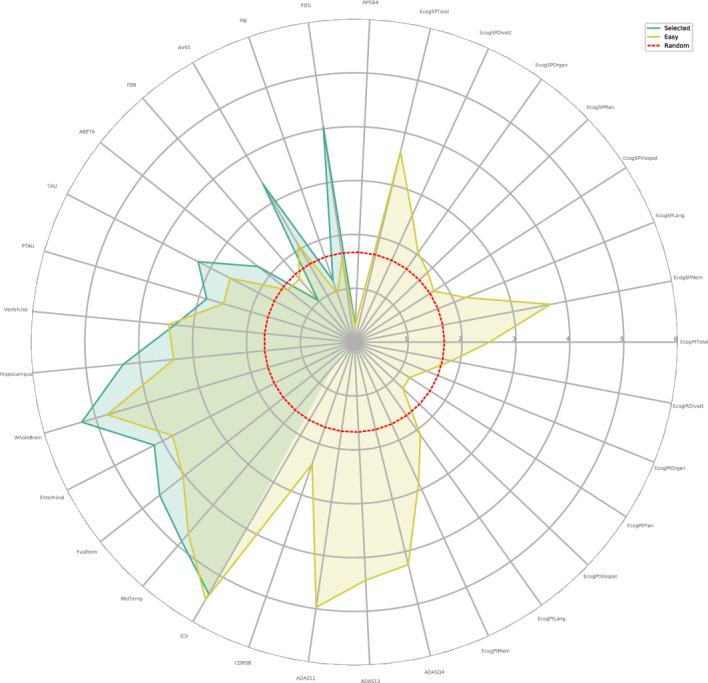
Results of unknown feature prediction experiment. The axis of the radar graph represents different features, and the length of the model on the axis represents the similarity between the predicted value and the real value. The red dotted line indicates the similarity between the random variable and the real value, the green indicates the effect of IPDM when the visible feature is Selected Features, and the yellow indicates the effect of IPDM when the visible feature is Easy Features.

Since the model does not know the value range of the unseen features, we normalize the real values and the predicted values of the entire test set with the following formula,

$$\begin{aligned} Normalize(H) = \frac{H-min\{H\}+\varepsilon }{max\{H\}-min\{H\}}, \end{aligned}$$

where *H* is the input parameters, $\varepsilon$ is a tiny offset, and we set $\varepsilon =10^{-5}$.

Then we take the multiplicative inverse of their KL divergence as the evaluation standard of prediction accuracy, where

$$\begin{aligned} D_{KL}(H_{pred}\Vert H_{real}) = \sum _xH_{pred}(x)\cdot log\frac{H_{pred}(x)}{H_{real}(x)}_. \end{aligned}$$

We disguise random variables as unseen features and enforce the IPDM to predict them as the baseline, and the results are shown in Fig. 12. For some unseen features, such as ICV, the similarity between the prediction and real values is high, but for features such as APOE4, the prediction accuracy is relatively poor. This indicates that IPDM can imitate the deep implicit connection between some features. In addition, it also explains the importance of APOE4 and other characteristics that are highly contributive and irreplaceable for the diagnosis and prediction of Alzheimer’s disease.

We believe that the application of IPDM in the auxiliary diagnosis and prediction of diseases, especially chronic diseases with unclear early-onset characteristics and sudden diseases with insufficient data, can reduce costs and help patients to detect, intervene and treat the diseases in time. In addition, IPDM saves the computational cost of reconstructive pre-training and accelerates the application of advanced methods of artificial intelligence in the medical field.

Nevertheless, there are still some problems to be solved. The use of pre-trained language models (PLMs) makes the time and space cost of fine-tuning significantly higher than that of the non-pre-training language models. Due to the use of the prior knowledge based on the pre-training corpus, some stereotypes may interfere with the model in the downstream task. With more application scenarios of PLMs, the processing of multi-modal mixed data will become a challenge.

Our future work will be mainly aimed to solve the challenges brought by multimodal data, and try to get a more perfect model structure that can integrate texts, structured EHRs, pictures and other multimodal data, so as to better assist disease diagnosis and prediction. Finally, establishing a stable and low-resource medical diagnostic infrastructure system via machine learning algorithm requires further considerable efforts from neuroscience, healthcare, biomedicine, and information science.

## Methods

### Datasets

Data of the Alzheimer’s disease diagnosis task and the Alzheimer’s disease progression prediction task used in this article were obtained from the Alzheimer’s Disease Neuroimaging Initiative (ADNI) database (adni.loni.usc.edu). And the data of ICU death prediction task is obtained from MIMIC-III database^45,46^.

#### Alzheimer’s disease diagnosis task

Data in the ADNI database is stored in structured electronic health records (EHRs), which contain the features to be studied and orther information such as patient number, test time, test number, diagnosis result and data source. When constructing the dataset, first remove irrelevant columns and only retain the subject number, test time, features and diagnosis results. Since the ADNI database includes the data of subjects from multiple countries and regions, there are records of different languages, for the convenience of processing, delete the data of languages with lower proportions such as Hawaiian. After completing the above data cleaning work, a total of 10,993 pieces of data were obtained. Next, according to the diagnostic results in the records, label each piece of data with CN, MCI and AD. Finally, randomly extract 2000 pieces of data as the test dataset, and the remaining 8993 pieces are used as the training dataset. In the training dataset, there are 3127 CNs, 3903 MCIs, and 1963 ADs; in the test dataset, there are 675 CNs, 895 MCIs, and 430 ADs.

#### Alzheimer’s disease progression prediction task

The data cleaning work for the Alzheimer’s disease progression prediction task is consistent with the Alzheimer’s disease diagnosis task. After the data cleaning, according to the subject number, the test records of the same subject in different periods were integrated, and then the subjects who were diagnosed with MCI at the initial test were screened. A total of 4798 pieces of data were obtained and the participants were deleted. Then delate data which recorded less than eight times, the remaining data are marked with KEEP or WORSE according to they are ended with MCI or AD. Finally, a total of 619 pieces of data were obtained, 80 pieces of data were extracted to construct the test dataset, and the remaining 539 pieces were used as the training dataset. Among them, the training dataset contains 224 KEEPs and 315 WORSEs, and the test dataset contains 28 KEEPs and 52 WORSEs.

#### ICU death prediction task

The dataset of ICU death prediction task was constructed from MIMIC-III database^38,44^. First, extract the hospitalization records and examination records of each patient according to the patient number, delete data missing the patient number and ICU file number, and obtain 3,431,622 test records of 42,276 patients. Because most features are at a missing rate of more than 70%, only diastolic/systolic/mean blood pressure, heart rate, respiratory rate and oxygen saturation are remained. Then, in the hospitalization records of each patient, extract “4+24” h of monitoring data, integrate the examination results of the first 4 h as the current status of the patient, and label the patient’s status with LIVE or DIE at the end of the next 24 h, and 143,183 data are obtained, including 107,139 LIVEs and 36,044 DIEs with a mortality rate of 25.17%. We splited the dataset refering to the standard of Harutyunyan et al.^43^ and finally obtained 118,922 pieces of training dataset, including 88,838 LIVEs and 30,084 DIEs, and 24,261 pieces of test dataset, including 18,301 LIVEs and 5960 DIEs.

### On ethical data use related to this manuscript

Ethics approval and consent to participate: As per ADNI protocols, all procedures performed in studies involving human participants were in accordance with the ethical standards of the institutional and/or national research committee and with the 1964 Helsinki declaration and its later amendments or comparable ethical standards. More details can be found at adni.loni.usc.edu. (This article does not contain any studies with human participants performed by any of the authors).

MIMIC-III database is available on PhysioNet repository, and researchers employing the MIMIC-III database are subject to the PhysioNet Credentialed Health Data Use Agreement 1.5.0 (https://physionet.org/content/mimiciii/view-dua/1.4/).

## Conclusion

Health and wellness are paramount to human survival and progress. However, the emergence of chronic diseases, characterized by subtle early signs and acute illnesses with limited data, poses a significant risk to human health. Despite advances in medical science and technology, which have resulted in a multitude of effective treatment plans for various diseases, we continue to grapple with some persistent health challenges. Diseases such as Alzheimer’s and critical conditions in intensive care units (ICUs) are particularly complex due to their subtle early symptoms and limited distinguishing features. Even in regions with abundant medical resources, the prohibitive costs of diagnostic tests can delay the detection of these diseases until they directly threaten the patient’s health. Unfortunately, this often occurs when the crucial window for intervention and treatment has passed. This underlines the necessity for cost-effective, early detection systems to accurately diagnose these conditions at the onset, ensuring timely intervention and significantly improving patient outcomes. To address the outlined challenges, we propose the Input Prompting and Discriminative language model with the Mixture-of-experts framework (IPDM). This approach leverages the pre-training knowledge of Pretrained Language Models (PLMs) and applies it to structured Electronic Health Records (EHRs) with minimal modifications for downstream tasks. Our approach aims to create a robust and resource-efficient medical diagnostic system through PLMs, offering a supportive analysis tool for medical experts. Initially, an input prompt, constructed with meta information, serves as the system input. The models within this system utilize a prompt-tuning method based on the discriminative pre-training language models. Finally, a gating network directs the weighted predictions from multiple models, identical in structure but differing in initialization, as the system output. We evaluated the effectiveness of IPDM by fine-tuning it for one disease diagnosis task and two disease prediction tasks: diagnosing Alzheimer’s disease, predicting Alzheimer’s disease progression, and predicting ICU mortality. Extensive experiments were performed using various machine learning models, including Logistic Regression, Support Vector Machine, Decision Tree, Random Forest, Multi-layer Perceptron, Convolutional Neural Network, and Long Short Term Memory, to ascertain IPDM’s effectiveness. We established a few-feature settings to test IPDM’s effectiveness in the context of subtle early symptoms and insufficient features. A few-sample setting was designed to assess IPDM’s performance under low-resource scenarios. We also conducted ablation experiments to verify the effectiveness of various IPDM improvements.

Our primary contributions are as follows: (1) IPDM successfully translates pre-training results from natural language pre-training corpus to structured EHRs with minimal fine-tuning alterations. It outperforms other leading machine learning methods in one disease diagnosis task and two disease prediction tasks. (2) Experimental results using a few-feature setting demonstrate that IPDM surpasses other machine-learning methods in diagnosing and predicting chronic diseases with subtle early symptoms and acute diseases with limited features. (3) Under low-resource scenarios, IPDM exhibits superior stability compared to other machine learning methods, as evidenced by our experimental results. (4) Ablation experiments affirm the effectiveness of various improvements within IPDM.

## Data Availability

The data that support this study are available from the Alzheimer’s Disease Neuroimaging Initiative (ADNI) database (adni.loni.usc.edu) and the MIMIC-III database^38^. Restrictions apply to the availability of ADNI database, which was used under license from the data provider. Data of MIMIC-III analyzed in this study is available on PhysioNet repository^46^.
